# Fabrication of Adhesive Resistance Surface with Low Wettability on Ti6Al4V Alloys by Electro-Brush Plating

**DOI:** 10.3390/mi10010064

**Published:** 2019-01-18

**Authors:** Xiaojuan Dong, Jianbing Meng, Haian Zhou, Rufeng Xu, Xue Bai, Haiyun Zhang

**Affiliations:** School of Mechanical Engineering, Shandong University of Technology, Zibo 255000, China; dongxiaojuan@sdut.edu.cn (X.D.); zhouhaian@sdut.edu.cn (H.Z.); xurufeng2003@126.com (R.X.); lz8016@126.com (X.B.); zhy@sdut.edu.cn (H.Z.)

**Keywords:** adhesive resistance, low wettability, Ti6Al4V alloys, electro-brush plating

## Abstract

Anti-adhesive Ni coatings with low wettability were successfully fabricated on Ti6Al4V substrates via an electro-brush plating method, and subsequently modified with a fluoroalkylsilane (FAS) film. The surface morphology, chemical compositions, and wettability of the as-prepared coatings were measured using scanning electron microscopy (SEM), X-ray diffractometer (XRD), Fourier transform infrared spectrophotometry (FTIR), and contact angle measurements. The results showed that the surface of Ti6Al4V substrate was endowed with flower-like structures. Each flower-like cluster was constituted by a large number of Ni ions. After surface modification of FAS, the as-prepared Ti6Al4V surface had a water contact angle as high as 151.5°, a sliding angle close to 2.1°, and a solid surface energy as low as 0.97 mJ/m^2^. Potentiodynamic polarization tests showed that the Ni coating could provide a stable corrosion protection. In addition, the effects of processing conditions, such as working voltage, relative velocity, electrolyte concentration, and processing time, were investigated. The mechanism of the adhesive resistance was proposed, and the low wettability of Ti6Al4V surfaces was explained by Cassie–Baxter model. As a result, it was necessary to reduce the fraction of the solid–liquid interface in order to achieve anti-adhesive surface.

## 1. Introduction

Titanium alloy has many attractive properties like outstanding mechanical and thermal properties, excellent corrosion resistance, and high strength-to-weight ratio, which make it to be one of the most widely used materials for successful application in the aerospace, automotive, and biomedical fields [1,2,3]. Recently, anti-adhesive surfaces on titanium alloys have attracted much attention. Various methods, such as anodizing, electrochemical etching, micro-arc oxidation, and so forth, have been developed to fabricate anti-adhesive surfaces with low wettability on titanium alloys. Gao et al. fabricated a low-wetting surface with a water contact angle of 158° as well as a sliding angle of 5.3° on Ti6Al4V alloy by anodization [4]. Shen et al. prepared an anti-adhesive surface with micro–nanoscale hierarchical structures on Ti6Al4V alloys through a combination of sand blasting and hydrothermal method. This process improved the water contact angle from 134° to 161°and the sliding angle from 12° to 3° [5]. Wang et al. selected Pb(CH_3_COO)_2_ as the immersion solution and fabricated low adhesive surfaces with a water contact angle of 165° and a sliding angle of 4.6° on Ti6Al4V substrates by means of immersion method [6]. Lu et al. developed a simple electrochemical etching method to fabricate anti-adhesive surfaces on titanium substrates [7]. The etched surfaces had a water contact angle above 150°, as well as a sliding angle of only 2°. Jiang et al. prepared a low-wetting surface with high corrosion resistance on biomedical Ti6Al4V substrates by micro-arc oxidation [8]. Therefore, the preparation of low-wetting surface on titanium alloy substrate can significantly improve the adhesion and corrosion resistance. However, most of these methods involved some drawbacks, including expensive equipment, strict conditions, and complex operation. In contrast, electro-brush plating is considered as an effective method to fabricate artificial anti-adhesive surface because of its low cost, high efficiency, and large working area. As far as we know, this method has been widely used in carbon steel, aluminum alloy, and stainless steel [9]. However, there are few reports on the adhesive resistance of titanium alloy surfaces prepared by electro-brush plating. In this study, a facile electro-brush plating method for preparing anti-adhesive surfaces of Ti6Al4V alloys was introduced. The processing conditions for fabricating Ti6Al4V surfaces with flower-like microstructures were measured, and a detailed evolution of surface morphologies was studied. Afterwards, the effects of processing parameters such as working voltage, relative velocity, electrolyte concentration, and processing time were investigated.

## 2. Materials and Methods

### 2.1. Materials

Ti6Al4V plates (Fe 0.3%, C 0.1%, N 0.05%, H 0.015%, O 0.20%, V 3.5~4.5%, Al 5.5~6.8%, and Ti 90.0%), sizing of 50 mm × 40 mm × 5 mm, were produced by Beijing AMC Powders Ltd. (Beijing, China). Abrasive papers (from 500 to 2000 grades) were bought from Guangzhou Shuncheng Abrasive Co., Ltd. (Guangzhou, China). All the other chemicals were of analytical grade, which were supplied by Sinopharm Group Chemical Reagent Co., Ltd. (Shanghai, China). As a low surface energy material, fluoroalkylsilane (FAS, C_8_F_13_H_4_Si(OCH_2_CH_3_)_3_) was purchased from Shanghai Sinofluoro Chemicals Co., Ltd. (Shanghai, China).

### 2.2. Experimental Procedure

Sample pre-treatment—Ti6Al4V sample surfaces were polished with abrasive papers until surfaces were shined to wipe off the titanium oxide layer, and rinsed ultrasonically with anhydrous ethanol and deionized water in turn for 15 min. Next, the specimens were soaked in 80 °C degreasing solution for 10 min. The acid washing and activation was performed for 6 min at room temperature. And then, a thin intermediate layer was formed on the substrate by electroless plating to avoid the sample re-oxidation. Electro-brush plating—the inside of the brush was made of cold-pressed graphite, and the outside was covered with degreased cotton. The brush and Ti6Al4V sample were connected to positive and negative of DC power supply, respectively. The pre-treated sample surface was immersed in the plating solution for 1–5 min at 60 °C using different brush plating voltages (ranging from 5 to 30 V). Finally, the sample surface was rinsed with deionized water. Fluorination modification 1.0 wt % fluoroalkylsilane–ethanol solution was prepared in a beaker and stirred with a magnetic stirrer at a speed of 150 r/min for 2 h. The as-prepared surface was immersed in 1.0 wt % ethanol solution of FAS for 90 min at ambient temperature, and heat-treated at 180 °C for about 1 h. Degreasing solution, washing solution, activation solution, and plating solution are listed in Table 1.

### 2.3. Characterization

The surface morphology of the as-prepared coatings was characterized using an optical microscope (Leica DVM2000, Leica Microsystems, Wetzlar, Germany) and a scanning electron microscope (SEM, Sirion 200, ThermoFisher Scientific, Hillsboro, OR, USA). The water contact angles were measured at ambient temperature using an optical contact angle measuring instrument (DSA 100, KRÜSS GmbH, Hamburg, Germany). The sliding angles were measured using the conventional sessile-drop method. A 5-μL deionized water droplet was dropped on the obtained surface, and the average of three measurements at different positions was regarded as the final contact angle. The angle at which the water droplet initiated to roll off the tilted surface was defined as water sliding angle. The surface chemical composition was investigated using a Fourier transform infrared spectrophotometer (FTIR, Nicolet 5700, Thermo Elecron Scientific Instruments Corp., Hillsboro, OR, USA) and an X-ray diffractometer system (XRD-6000, Shimadzu, Kyoto, Japan). In addition, the corrosion resistance of the Ti6Al4V specimen was examined with the changes of the corrosion potential and corrosion current density, which were measured using an electrochemical workstation (CHI660E, CH Instruments, Austin, TX, USA).

## 3. Results and Discussion

### 3.1. Surface Morphology

**Figure 1:**
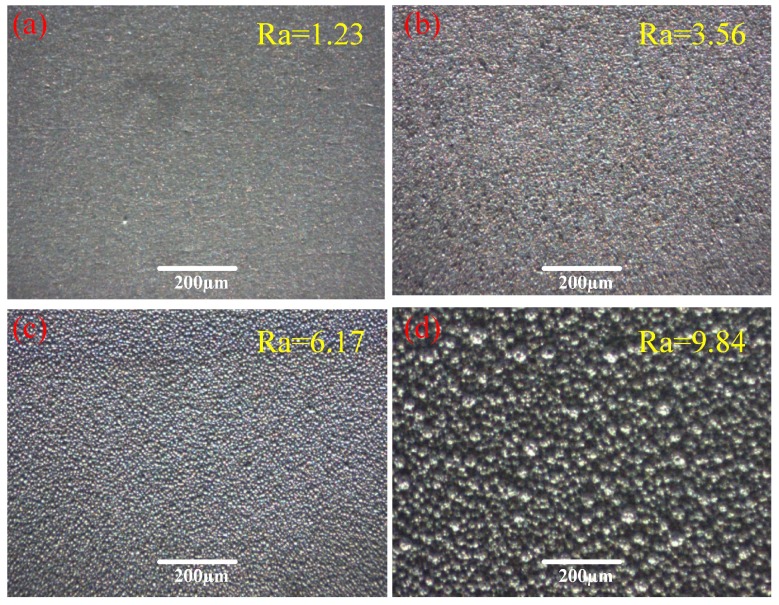
Photos of prepared surfaces obtained in the concentration of 400 g/L at the voltage of 25 V, the relative velocity of 7 m/min and different processing times—(**a**) 1 min, (**b**) 3 min, (**c**) 5 min, and (**d**) 7 min.

To understand the growth process of morphological structure on as-prepared Ti6Al4V substrate surface, the effect of the processing time on the surface morphology was studied by SEM, and observations were done at the working voltage of 25 V, the relative velocity of 7 m/min, and the NiSO_4_ concentration of 400 g/L. Figure 1 shows the optical photos of the coating constructed on Ti6Al4V substrate for different plating times. In Figure 1a,b, some micro-mastoid structures appear and are unevenly distributed on the coating. When the processing time is 5 min, the coating is uniformly and compactly covered with papillary structures, as shown in Figure 1c. Meanwhile, relatively finer rough structures formed on the mastoids, leading to increased roughness of sample surfaces. Surface roughness measurements showed that the roughness of Ra of the Ti6Al4V surfaces increased from 1.23 at 1 min to 3.56 at 3 min, 6.17 at 5 min, and 9.84 at 7 min. These rough structures were necessary in low-wetting surfaces. However, it can be observed from Figure 1d that these microstructures are joined with each other and assembled into interconnected block-like structures, when the plating time reached 7 min. Thus, the diameter of these microstructures had become larger and nanometer structures disappeared. Consequently, the lack of rough micro/nanometer structures resulted in the decrease of the low wettability.

**Figure 2:**
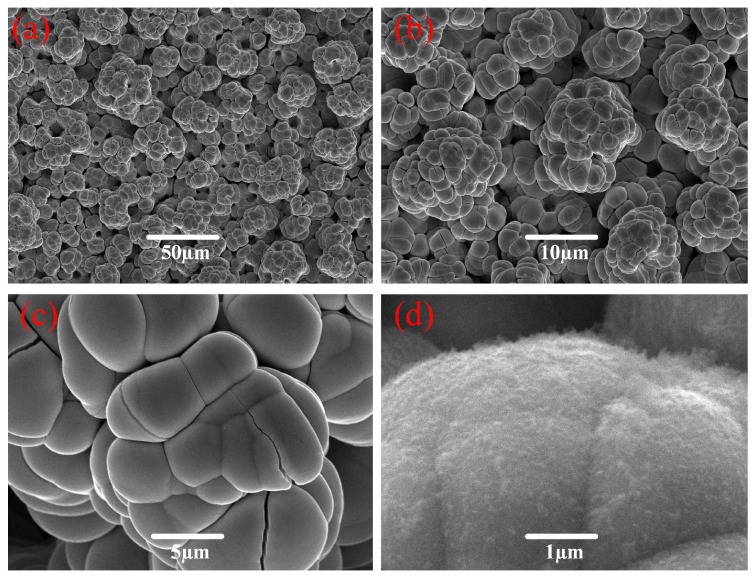
Scanning electron microscopy (SEM) images of Ti6Al4V alloy surfaces obtained in the concentration of 400 g/L at the voltage of 25 V, the relative velocity of 7 m/min, and the processing time of 5 min with different magnifications—(**a**) 500×, (**b**) 1000×, (**c**) 5000×, and (**d**) 20,000×.

Figure 2 shows the SEM images of the obtained coating fabricated by the electro-brush plating for 5 min in the NiSO_4_ concentration of 400 g/L, at the working voltage of 25 V, and the relative velocity of 7 m/min with different magnifications. In Figure 2a,c, some micro cones with an average diameter of about 10 μm assemble into an interconnected porous surface. Furthermore, flower-like microstructures consisting of cones are compactly distributed over the Ti6Al4V surface, as shown in Figure 2b. In the high-resolution image (Figure 2d), there exist many nano flosses on these micro cones. These nano flosses will contribute to the formation of a hierarchical structure. Therefore, the above results suggest that the rough hierarchical structure is of great significance for achieving anti-adhesive and low-wetting properties.

### 3.2. Surface Wettability

Three classical wetting models on the rough surface were developed by Yong, Wenzel, and Cassie [10], respectively. According to the wetting behavior of the as-prepared Ti6Al4V surface, instead of infiltrating into the rough hierarchical structures, the water droplet suspended on the air-filled Ni deposit, which can be interpreted by the Cassie theory [11,12]. Furthermore, it can be also proved that the water droplets on the as-prepared rough structure are consistent with the Cassie–Baxter model by the general wetting diagram [7], which is given by the following equation.

(1) $$\cos\theta_{c}=f\left(\cos\theta +1\right)-1$$

where *θ_c_* is the water contact angle of the as-prepared surface, *θ* is the contact angle on the untreated surface, and *f* is an area surface fraction defined as the ratio of wetted surface area to total surface area.

In Cassie’s model, air pockets were trapped under the liquid, leading to a liquid–solid/liquid–gas composite interface. The area fraction is represented by *f*, where the liquid is in contact with a solid surface as opposed to air, which can be obtained from Equation (1). In the electro-brush plating process, working voltage, plating time, relative velocity, and NiSO_4_ concentration play important roles on the wettability, where the water contact angle *θ_c_* and the solid area fraction *f* are used as evaluation indexes.

**Figure 3:**
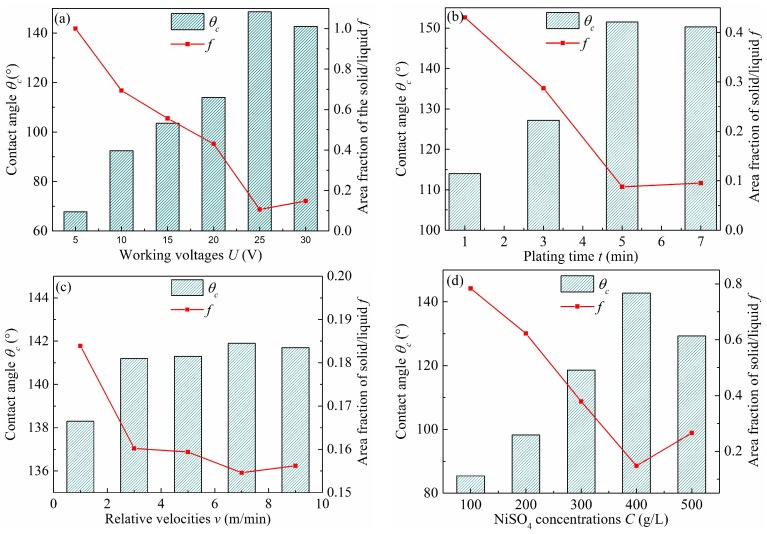
Contact angles and solid–liquid area fraction of Ti6Al4V alloy surfaces at different process parameters, such as (**a**) working voltage, (**b**) plating time, (**c**) relative velocity, and (**d**) NiSO_4_ concentration.

The electro-brush plating process was carried out at different working voltages ranging from 5 to 30 V. The result is shown in Figure 3a. It can be found that the water contact angle on the Ti6Al4V substrate gradually increased from 67.8° to 148.6° with the increase of the working voltage from 5 to 25 V, and then decreased evidently to 142.7°. Compared with the water contact angle, the solid area fraction presented the opposite trend. This result showed that the Ti6Al4V substrate obtained a better low wettability with the water contact angle of 148.6° and the solid area fraction of 0.106, when the working voltage was 25 V.

Interestingly, the increase of the electro-brush plating time resulted in the transformation of surface wettability. Figure 3b shows the effect of processing time on the water contact angle and solid area fraction when the working voltage was 25 V, the relative velocity was 7 m/min, and the NiSO_4_ concentration was 400 g/L. In Figure 3b, the water contact angle markedly increased with extended plating time, and reached the highest value of 151.2° after 5 min, and then decreased slightly but remained above 150°. Meanwhile, it can be also found that the solid area fraction decreased greatly to 0.088 when the plating time reached 5 min. After 5 min, the solid area fraction increased slightly to 0.095. Consequently, the above result suggested that the wettability of the as-prepared surface switched from hydrophilic to hydrophobic or even super hydrophobic.

In addition, the relative velocity has a great importance on the wettability of the as-prepared Ti6Al4V surface. The Ti6Al4V substrate was electro-brush plated for 3 min in the 400 g/L NiSO_4_ solution at 20 V, where the relative velocity ranged from 1 to 9 m/min. The result is shown in Figure 3c. The water contact angle was only 138.3°, when the relative velocity was 1 m/min. As the relative velocity increased to 5 m/min, the water contact angle significantly increased to 141.3°. Below the range 5–7 m/min, it reached the highest value of 142°. However, when the relative velocity exceeded 7 m/min, the water contact angle decreased evidently to 141.7°. Compared with the water contact angle, the change of the solid area fraction was opposite. In fact, the lower relative velocity, corresponding to longer plating time, resulted in coarse grains, lower porosity, and poor hierarchical structures. In contrast, under the higher relative velocity, the reaction time was very short, which limited the deposition of Ni ions and the growth of the hydrophobic coating.

The effect of concentration of Ni ion on the water contact angle and solid area fraction was also investigated at 20 V of working voltage and 7 m/min of relative velocity for 5 min of plating time. The results are shown in Figure 3d. Varying the Ni ion concentration from 100 to 400 g/L, the water contact angle significantly increased from 85.4° to 142.8°, and the solid area fraction decreased from 0.784 to 0.148. Actually, when the concentration of Ni ion was relatively low (100 g/L), the hierarchical structure composed of the micro cone and nano floss had not yet been formed. Moreover, when the concentration of Ni ion was higher (500 g/L), the porosity decreased and the flower-like hierarchical structure was not obvious. Consequently, the above morphologies hardly provided enough micro/nano structures to make Ti6Al4V surfaces low-wetting.

### 3.3. Surface Adhesive Resistance

Anti-adhesive ability is an important application for the low-wetting surface. Generally, solid surface energy and sliding angle are two key factors affecting surface adhesion [13,14]. The lower the solid surface energy is, the lower the surface adhesion force. There are several methods for calculating solid surface energy [15]. In this study, derived by Neumann et al. [16], the solid surface energy can be obtained as:

(2) $$\cos\theta =-1+2\sqrt{\frac{\gamma_{S}}{\gamma_{L}}}\exp\left\{-\beta {\left(\gamma_{L}-\gamma_{S}\right)}^{2}\right\},$$

where *θ* is the measured contact angle, *γ_S_* is the solid energy, *γ_L_* is the liquid surface energy, and *β* is a constant (*β* = 1.247 × 10^−4^ m^2^/mJ). Deriving on both sides of the equation, Equation (2) can be written as:

(3) $$\ln\gamma_{S}-\ln\left\{\gamma_{L}\cos^{4}\left(\frac{\theta }{2}\right)\right\}=2.494\times 10^{-4}{\left(\gamma_{L}-\gamma_{S}\right)}^{2}.$$

It is obvious that each side in Equation (3) has an unknown value. However, the liquid used here is deionized water, and the liquid energy *γ_L_* is 72.8 mJ/m^2^ [17]. The measured contact angles and the water surface energy were introduced into Equation (3), and two curves were drawn. Thus, the intersection point was considered as the solid surface energy.

**Figure 4:**
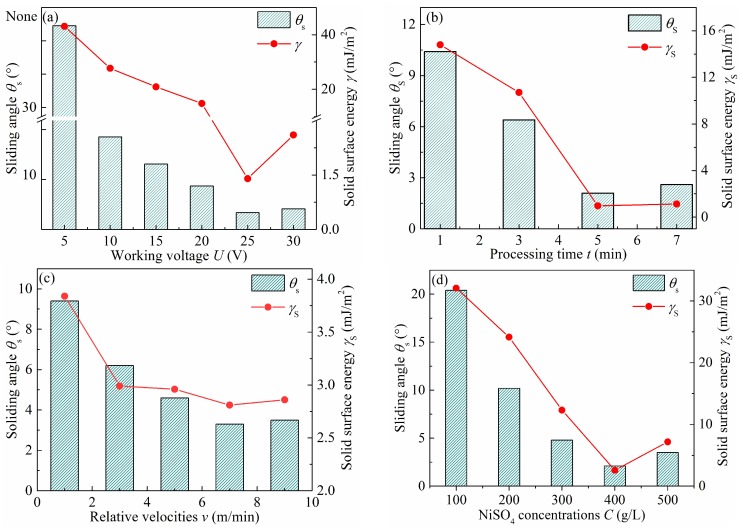
Sliding angles and solid surface energy of Ti6Al4V alloy surfaces at different process parameters, such as (**a**) working voltage, (**b**) plating time, (**c**) relative velocity, and (**d**) NiSO_4_ concentration.

The effects of the working voltage, processing time, relative velocity, and NiSO_4_ concentration on surface adhesive resistance were investigated in detail. Figure 4a shows the relationship of the working voltage with the solid surface energy and the sliding angles. The Ti6Al4V surface was electro-brush plated for 5 min in the 400 g/L NiSO_4_ solution at 5 m/min relative velocity. The working voltage ranged from 5 to 30 V. The solid surface energy decreased with the increase of the working voltage. This can be explained by the changes in surface morphology. With the increase in the working voltage, the micro-mastoid increased, and the contact area of liquid–solid interface reduced. Considering that solid surface energy depends on the contact area, the variation in contact area is a major factor affecting the adhesion force. Consequently, the more the working voltage is, the smaller the solid surface energy, and the lower the surface adhesive force. In terms of the sliding angles, the variation trend of the water sliding angles with the working voltage is similar to that of the solid surface energy.

In addition, the plating time has a great influence on the adhesive resistance of the as-prepared Ti6Al4V surface. Figure 4b shows the effect of processing time on the solid surface energy and sliding angle when the working voltage was 25 V, the relative velocity was 7 m/min, and the NiSO_4_ concentration was 400 g/L. It can be found in Figure 4b that the solid surface energy and sliding angle markedly decreases with the extended plating time, and reaches the minimum value of 0.97 mJ/m^2^ and 2.1° after 5 min, then increases slightly to 1.13 mJ/m^2^ and 2.6° when the plating time is 7 min.

Relative velocity is another important processing parameter in the electro-brush plating process. Figure 4c shows the relationship of the relative velocity with the solid surface energy and the sliding angle. The Ti6Al4V surface was electro-brush plated for 3 min in the 400 g/L NiSO_4_ solution at 20 V. In Figure 4c, the solid surface and the sliding angle gradually decrease from 3.84 mJ/m^2^ and 9.4° to 2.81 mJ/m^2^ and 3.3° with the increase of the relative velocity from 1 to 7 m/min, and then increase evidently to 2.86 mJ/m^2^ and 3.5° when the relative velocity is 9 m/min.

The influence of the concentration of Ni ion on the adhesive resistance of the Ti6Al4V substrate was also investigated at 20 V of working voltage and 7 m/min of relative velocity for 5 min of plating time. From Figure 4d, it can be seen that the solid surface energy decreases with the extension of the concentration of NiSO_4_. The solid surface energy reaches the lower value of 2.58 mJ/m^2^ when the NiSO_4_ concentration is 400 g/L. In addition, Figure 4d shows that the as-prepared Ti6Al4V surface has a lowest sliding angle of 3.4°, allowing the droplet to roll off with ease. The results show that the water droplet does not penetrate into the hierarchical structure, but rather is suspended on the top of the as-prepared surface. Therefore, it is considered that low solid surface energy and small sliding angle play an important role in improving the surface adhesive resistance.

### 3.4. Chemical Composition

**Figure 5:**
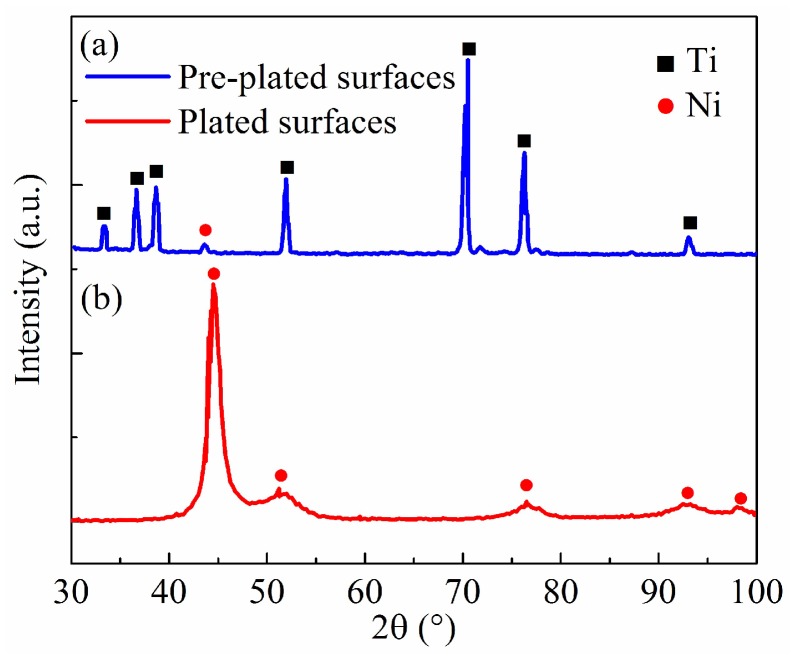
X-ray diffractometer (XRD) patterns of the (**a**) pre-plated and (**b**) plated surfaces via electro-brush plating.

The crystal structures of pre-plated and plated Ti6Al4V surfaces were investigated by performing XRD patterns, as shown in Figure 5. In the 2*θ* scan range from 30° to 100°, the pre-plated surface showed characteristic peaks of Ti(100), Ti(002), Ti(101), Ti(102), Ti(103), Ti(112), and Ti(104) at 35.05°, 38.35°, 40.29°, 51.95°, 70.25°, 76.35°, and 93.05°, respectively. These Ti peaks are in agreement with the Ti crystallographic data, which are attributed to the original Ti6Al4V substrates. Meanwhile, it can be found that there exists a peak at around 45° in Figure 5a, corresponding to Ni crystallographic data. It indicates that the Ni deposits begin to appear on the sample surface. Several different peaks at 44.51°, 51.24°, 76.51°, 92.49°, and 97.98° are in accordance with Ni(11), Ni(200), Ni(220), Ni(311), and Ni(222), respectively, as shown in Figure 5b. It is clear that the Ni coating is successfully formed on the Ti6Al4V substrates after electro-brush plating process. Compared with Figure 5a, there are only Ni peaks and no Ti peaks in Figure 5b. It confirms that the Ti6Al4V surface has been completely covered by Ni deposits.

**Figure 6:**
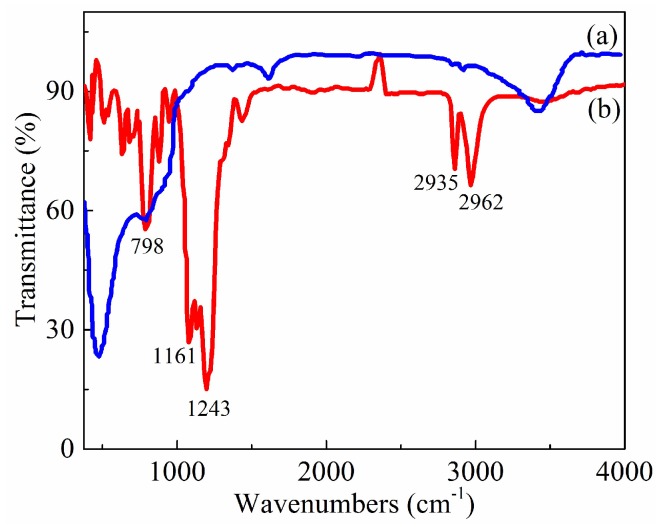
Fourier transform infrared spectrophotometry (FTIR) spectrums of Ti6Al4V surfaces fabricated via electro-brush plating (**a**) before fluoroalkylsilane (FAS) modification and (**b**) after FAS modification.

Generally, surface chemical compositions are investigated with the FTIR spectrum. Owing to its excellent low surface energy, FAS is widely used to modify hierarchical structures for improving the low wettability and anti-adhesion property of the solid surface. Figure 6 shows the FTIR spectrums of Ni-plated Ti6Al4V surfaces before and after FAS modification. After FAS modification, the absorption peak of the C–F bond appeared at around 1161 cm^−1^ while that for the CF_2_ bond at around 1243 cm^−1^. The two absorption bands at around 2935 and 2962 cm^−1^ were assigned to the C–H stretching vibration of the –CH_3_ and –CH_2_– groups, which resulted in the decrease of the surface energy. Obviously, FAS molecules were successfully self-assembled on Ti6Al4V surfaces with hierarchical structures. The reason was that there were Si–OR polar groups and a long hydrophobic chain in FAS. Si–OR groups of FAS in ethanol were easily converted to Si–OH groups. These new groups formed covalent bonds with TiO_2_ molecules of specimen surfaces and generated a layer of low-wetting molecular membrane. Additionally, there was a dehydration condensation reaction to form the hydrogen bond-driven binding between the hierarchical structure surface and FAS.

### 3.5. Surface Corrosion Resistance

**Figure 7:**
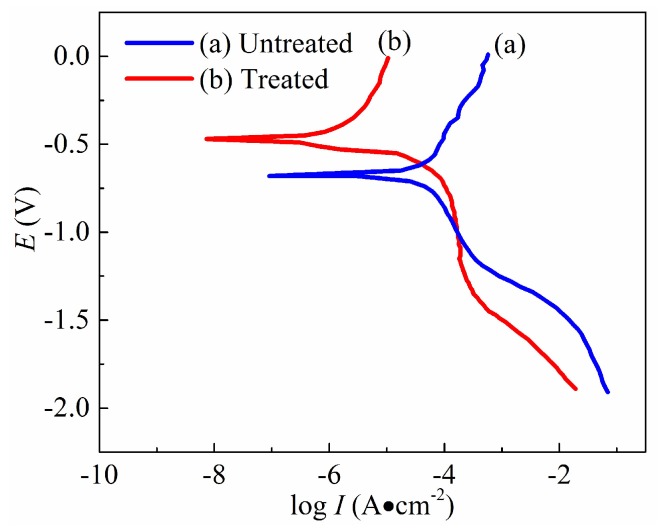
Potentiodynamic polarization curves of the (**a**) untreated and (**b**) treated Ti6Al4V surfaces.

**Figure 8:**
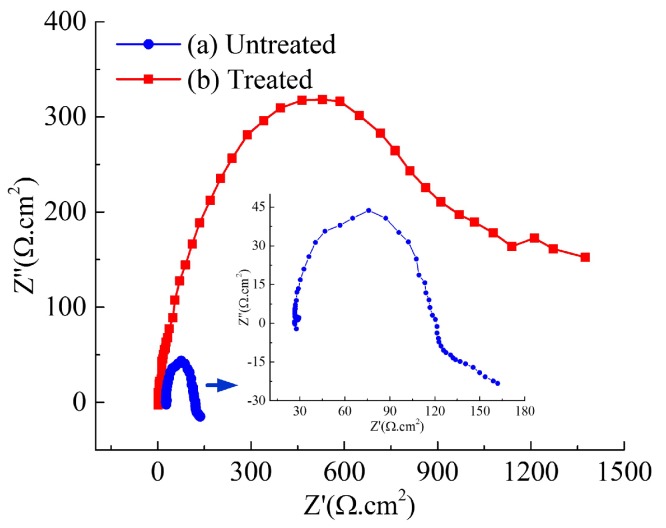
Nyquist plots of the (**a**) untreated and (**b**) treated Ti6Al4V surfaces in 3.5 wt % NaCl aqueous solution.

It is well known for the polarization curve to evaluate the corrosion resistance of a metal substrate. In fact, a higher corrosion potential or a lower corrosion current density corresponds to a better corrosion resistance and a lower corrosion rate. The potentiodynamic polarization curves of untreated and as-prepared Ti6Al4V surfaces for 5 min were obtained using Hank’s solution at the 25 V working voltage and the 7 m/min relative velocity in the 400 g/L NiSO_4_ concentration. The results are shown in Figure 7. Corrosion resistance of the as-prepared Ti6Al4V surface is greatly improved, compared with the unplated surface. It is clear that the whole polarization curve of the plated substrate surface has a shift towards the region of higher potential and lower current density. The values of corrosion current density and potential are 4.2 × 10^−7^ A/cm^2^ and −0.47 V for the Ni-plated specimen surface, and 2.47 × 10^−5^ A/cm^2^ and −0.68 V for the unplated alloy surface, respectively. Especially, the corrosion current density of Ni-plated surface decreased by more than 2 orders of magnitude compared with that of the unplated surface. Such low current density indicated a good corrosion resistance for the treated surface by electro-brush plating. Meanwhile, the corrosion potential of the Ni-plated Ti6Al4V surface was shifted in the noble direction by 210 mV relative to the unplated specimen surface. The corrosion potential of the treated sample surface was more positive than that of the untreated Ti6Al4V surface. The shift of the corrosion potential in the positive direction had an improvement in the protective properties of the anti-adhesive and low-wetting surface. In addition, the impedance spectroscopy measurement for corrosion behaviors were carried out in 3.5 wt % NaCl solution using a potentiostat with a potential range from −50 V to 400 V. Figure 8 shows that the impedance of the as-prepared sample surface is much higher than that of the untreated Ti6Al4V surface. The corrosion resistance mechanism of low-wetting surfaces proceeds as follows—when immersed in a corrosion solution, low-wetting surfaces composed of hierarchal rough structures can easily trap a large amount of air within the valleys between the rough structures. These “air valleys” then prevent the migration of corrosive ions. Consequently, these above results show that the anti-adhesive and low-wetting surface has outstanding corrosion resistance.

## 4. Conclusions

Anti-adhesive Ti6Al4V surfaces with a flower-like hierarchical structure were fabricated first via a simple electro-brush plating method and then followed by the surface modification with FAS. The optimal processing conditions for improving the adhesive resistance of the as-prepared surface have been obtained, i.e., 25 V of working voltage, 5 min of plating time, 7 m/min of relative velocity, and 400 g/L of NiSO_4_ concentration. The as-prepared surface has a better hydrophobicity with 151.5° of water contact angle and 0.088 of solid area fraction under the above-mentioned optimal processing parameters. Further, the as-prepared surface has pleasurable adhesive resistance with 0.97 mJ/m^2^ of solid surface energy and 2.1° of sliding angle. In addition, the corrosion resistance of the as-prepared Ti6Al4V surfaces was investigated. The results reveal that anti-adhesive surfaces can provide a favorable and stable protection for the Ti6Al4V alloys. The proposed method is feasible and of low cost. It can be widely applied for micro/nano devices or systems of low-wetting surfaces with good adhesive and corrosion resistance.

## Figures and Tables

**Table 1:** Composition and processing parameters of degreasing, washing, activation, and plating solutions.

Solution	Composition	Value	pH	Temperature (°C)	Time (min)
Degreasing Solution	Na_2_CO_3_	30 g/L	12	80	10
	NaOH	35 g/L			
	Na_3_PO_4_∙12H_2_O	30 g/L			
	Na_2_SiO_3_	5 g/L			
Washing Solution	HF (40%)	20 mL/L	0.6	25	1
	HCl (36%)	50 mL/L			
Activation Solution	HF (40%)	50 mL/L	4	25	5
	HNO_3_ (65%)	50 mL/L			
	H_2_O_2_ (30%)	100 mL/L			
Pre-plating Solution	NiSO_4_∙6H_2_O	40 g/L	5	25	10
	NaH_2_PO_2_∙H_2_O	20 g/L			
	CH_3_COONa	10 g/L			
	NH_4_Cl	10 g/L			
Plating Solution	NiSO_4_∙6H_2_O	100–500 g/L	3–4	60	1–5
	NiCl_2_∙6H_2_O	40 g/L			
	Na_3_C_6_H_5_O_7_·2H_2_O	15 g/L			
	C_6_H_11_NaO_7_	12 g/L			
	H_3_BO_3_	35 g/L			
